# Probiotic Therapy with *Lactobacillus acidophilus* and *Bifidobacterium animalis* subsp. *lactis* Results in Infarct Size Limitation in Rats with Obesity and Chemically Induced Colitis

**DOI:** 10.3390/microorganisms10112293

**Published:** 2022-11-18

**Authors:** Yury Y. Borshchev, Inessa Y. Burovenko, Alena B. Karaseva, Sarkis M. Minasian, Egor S. Protsak, Victor Y. Borshchev, Natalia Y. Semenova, Olga V. Borshcheva, Alexander N. Suvorov, Michael M. Galagudza

**Affiliations:** 1 Research Department of Toxicology, Institute of Experimental Medicine, Almazov National Medical Research Centre, 43 Dolgoozernaya Street, 197371 Saint Petersburg, Russia; 2 Department of Molecular Microbiology, Institute of Experimental Medicine, 12 Academic Pavlov’s Street, 197376 Saint Petersburg, Russia; 3 Department of Microcirculation and Myocardial Metabolism, Institute of Experimental Medicine, Almazov National Medical Research Centre, 15B Parkhomenko Street, 194021 Saint Petersburg, Russia; 4 Department of Pathophysiology with Clinical Pathophysiology Course, Pavlov First Saint Petersburg State Medical University, 6–8 Lev Tolstoy Street, 197022 Saint Petersburg, Russia; 5 Research Department of Pathology, Institute of Experimental Medicine, Almazov National Medical Research Centre, 43 Dolgoozernaya Street, 197371 Saint Petersburg, Russia; 6 Department of Fundamental Problems of Medicine and Medical Technologies, Saint Petersburg State University, 7/9 Universitetskaya Embankment, 199034 Saint Petersburg, Russia

**Keywords:** probiotics, infarct size, *Lactobacillus acidophilus (LA–5)*, *Bifidobacterium animalis* subsp. *lactis (BB–12)*, cytokines, lipopolysaccharide, short chain fatty acids

## Abstract

In this study, we investigated the effect of three different probiotics, namely, a combination of *Lactobacillus acidophilus (LA–5)* and *Bifidobacterium animalis* subsp. *lactis (BB–12)*, *Saccharomyces boulardii*, and *Enterococcus faecium L3* on myocardial infarct size in rats with diet-induced obesity (DIO) and chemically-induced colitis (CIC). Potential associations between the effects of probiotics on myocardial ischemia-reperfusion injury and gut microbiome patterns as well as the serum levels of pro- and anti-inflammatory cytokines, lipopolysaccharide, and short chain fatty acids were also studied. Intragastric administration of lyophilized *Lactobacillus acidophilus* and *Bifidobacterium animalis* subsp. *lactis* at a dose of 1.2 × 10^8^ CFU/mL for 15 days resulted in myocardial infarct size reduction in rats with DIO, CIC, and antibiotic-induced dysbiosis. This cardioprotective effect was associated with specific changes in cytokine concentrations, namely reduced levels of IL–1β, TNF–α, IL–2, and IL–8. At the same time, the use of *Lactobacillus acidophilus* and *Bifidobacterium animalis* subsp. *lactis* was accompanied by a significant reduction in lipopolysaccharide level, suggesting normalization of intestinal epithelial barrier permeability. However, the cardioprotective effect of *Lactobacillus acidophilus* and *Bifidobacterium animalis* subsp. *lactis* is not secondary to improved healing of the intestinal mucosa in CIC, as evidenced by the lack of difference in histopathological scores.

## 1. Introduction

Ischemic heart disease (IHD) remains to be the leading cause of death in the majority of populations worldwide. In the United States only, about 20.1 million adults aged 20 years and older have IHD, which is 7.2% of the total population [1]. Early myocardial revascularization is currently the only effective way to prevent the progression of ischemic myocardial injury in the patients with acute coronary syndrome (ACS). Although myocardial reperfusion results in significant improvement in clinical outcomes, approximately 25% of patients with timely revascularization have an inappropriately low index of myocardial viability [2], thereby necessitating the search for new therapeutic strategies aiming at the reduction in myocardial ischemia-reperfusion injury (IRI).

The general connection between gut microbiota and cardiovascular disease has been widely explored in the literature (for review, see [3] and references therein). Over the last decade, evidence has started to accumulate that the manipulation of gut microbiota might result in the altered sensitivity of myocardium to IRI. Both antimicrobial agents and probiotics could be used as tools for producing the potentially cardioprotective microbial pattern of the gastrointestinal tract. In their pioneering work, Lam V. et al. showed that the administration of vancomycin and probiotic strain *L. plantarum 299V* resulted in infarct size limitation in rats by 27 and 29%, respectively [4]. Later, the same group presented evidence for the cardioprotective effect of vancomycin or a mixture of other antimicrobial agents (streptomycin, neomycin, bacitracin, and polymyxin B) using an isolated rat heart subjected to global ischemia-reperfusion [5]. Our data indicate that tetracycline administration caused infarct size reduction in healthy rats with the reversal of the cardioprotective effect in the animals with obesity and inflammation of the colon [6]. The use of *Lactobacillus rhamnosus GR–1* in rats with permanent coronary artery occlusion resulted in attenuation of myocardial hypertrophy and improvement in both systolic and diastolic left ventricular function, which was associated with normalization of elevated blood level of leptin [7]. In 2017, Danilo C. A. et al. demonstrated that the administration of *Bifidobacterium animalis* subsp. *lactis 420 (B420)* caused a reduction in myocardial infarct (MI) size in mice with high fat diet-induced intestinal dysbiosis [8]. An Iranian group demonstrated that treatment with probiotic strains *Bifidobacterium breve*,* Lactobacillus casei*,* Lactobacillus bulgaricus* and *Lactobacillus acidophilus* for 14 days resulted in attenuation of myocardial injury in the isoproterenol-induced MI [9]. In some studies, the intestinal microbiome was manipulated with non-pharmacological approaches. For example, increasing physical exercise over the period of 4 weeks has been associated with considerable changes in the composition of gut flora, which was accompanied by increased post-MI stroke volume and left ventricular systolic function in mice [10]. At present, very little is known about the molecular mechanisms responsible for cardiac protection in altered microbiome states. Recent evidence indicated that the link between microbiota and cardioprotective pathways in the heart may be provided by certain bacterial metabolites, such as short-chain fatty acids (SCFAs). One of the main SCFAs, propionate, has been recently implicated as a key mediator of cardioprotection, as evidenced by its ability to reduce angiotensin II-mediated infarct size enlargement through G-protein coupled receptor 41 [11]. Interestingly, complete ablation of intestinal microbiota with intensive antibiotic treatment resulted in a reduction in infarct size in mice, leading to the conclusion that microbiota aggravates myocardial IRI by means of stimulating formation of neutrophil extracellular traps [12]. Collectively, these findings provide an experimental rationale for a new concept of myocardial protection against IRI via the modulation of intestinal microbiome composition. To achieve this goal, the microbiome could be manipulated either via its supplementation with certain “cardioprotective” strains in the form of probiotics or by eliminating its components interfering with cardioprotection using selective antimicrobial agents. However, our current understanding of the effects of intestinal microbiota on cardioprotective pathways is far from complete. In addition, it should be noted that all experimental data on the existence of probiotic-induced cardioprotection have been obtained using intact young rodents. This is in sharp contrast with the clinical realm, where a majority of patients experiencing ACS have multiple comorbidities. For example, it is known that more than 80% of patients with IHD are overweight or obese [13]. Such common comorbidities as obesity, diabetes mellitus type 2, and atherogenic dyslipidemia are known to reduce the effectiveness of cardioprotective interventions, which is considered to be a major translational barrier in the field [14]. Visceral obesity is associated with systemic low-grade inflammation, manifesting by mild to moderate elevation of proinflammatory cytokines in the blood [15], which is associated with myocardial dysfunction and attenuated cardioprotection [16]. At the same time, the effects of probiotics on myocardial tolerance to IRI in the animal models of comorbidity have not been evaluated before. Therefore, we were interested to study the effect of three different probiotics, namely, a combination of *Lactobacillus acidophilus (LA–5)* and *Bifidobacterium animalis* subsp. *lactis (BB–12), Saccharomyces boulardii*, and *Enterococcus faecium L3* on myocardial infarct size in rats with diet-induced obesity (DIO) and chemically induced colitis (CIC). The latter condition has been previously validated by our group as a model of systemic inflammatory response syndrome (SIRS) [6]. Potential associations between the effects of probiotics on myocardial IRI and gut microbiome patterns as well as the serum levels of pro- and anti-inflammatory cytokines, lipopolysaccharide (LPS), and SCFAs were also investigated.

Our data have corroborated aggravation of myocardial IRI in case of combination of DIO and CIC compared to the controls. Administration of *Lactobacillus acidophilus (LA–5)* and *Bifidobacterium animalis* subsp. *lactis (BB–12)* but not *Saccharomyces boulardii* or *Enterococcus faecium L3* resulted in a significantly smaller infarct size in the group of animals with comorbidities. This cardioprotective effect was associated with specific changes in gut microbiota and cytokine concentrations.

## 2. Materials and Methods

### 2.1. Ethics

All experiments were carried out in accordance with the European Convention for the Protection of Vertebrate Animals used for Experimental and other Scientific Purposes and the Guide for the Care and Use of Laboratory Animals (NIH publication No. 85–23, revised 1996). The Institutional Animal Care and Use Committee at the Almazov National Medical Research Centre approved the detailed study design (protocol # 17–DI–1; 30 May 2017). All procedures complied with the ARRIVE guidelines (http://www.nc3rs.org/ARRIVE (accessed on 1 November 2022)). All efforts were made to protect the animals and minimize their suffering during the study.

### 2.2. Animals

Male Wistar rats weighting 335 ± 15 g were taken for the study. The animals were maintained in individually ventilated cages under a 12:12 (light:darkness) circadian cycle and had free access to water and food.

### 2.3. Chemicals

All chemicals and reagents used in the study were purchased from Sigma-Aldrich (St. Louis, MO, USA) and were of analytical grade unless otherwise specified.

### 2.4. Modelling of Diet-Induced Obesity

DIO was induced in the animals by feeding them a high-carbohydrate, high-fat diet (HFCD; 45 % sucrose, 40 % fat; Dyets Inc., Bethlehem, PA, USA) for 5 weeks before isolation of the heart and modelling of global ischemia-reperfusion [17].

### 2.5. Induction of Colitis

Colitis was produced in obese rats via a single rectal administration of 3 % acetic acid at a volume of 2 mL using a polyethylene tubing (diameter, 2 mm), which was inserted to a depth of 5.5 cm into the rectum [18]. The rats were positioned for the hind limbs to be elevated above the head during the rectal administration and for 1 min afterwards to avoid leakage of the acetate solution.

### 2.6. Experimental Design

**Figure 1:**
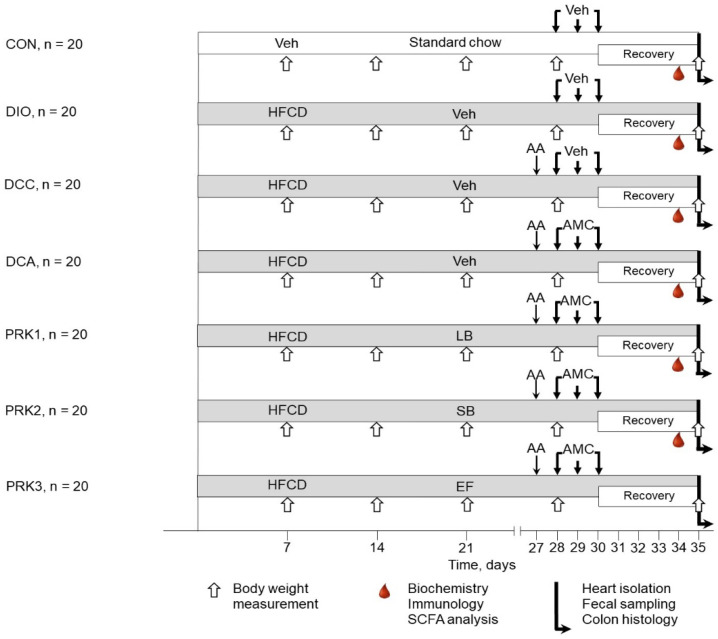
Experimental protocol. For details, see text. AA—acetic acid; AMC—antimicrobial compounds; EF—*Enterococcus faecium L3*; HFCD—high-fat, high-carbohydrate diet; LB—*Lactobacillus acidophilus (LA–5)* and *Bifidobacterium animalis* subsp. *lactis (BB–12)*; SCFA—short-chain fatty acids; SB—*Saccharomyces boulardii*; Veh—vehicle.

The animals were randomized into one of the following seven groups (Figure 1): control (CON, *n* = 20, each rat received 1 mL of the phosphate buffer); DIO (*n* = 20, rats with DIO were treated with the phosphate buffer); DCC (*n* = 20, rats with DIO and CIC were treated with the vehicle); DCA (*n* = 20, a day after CIC induction, rats with DIO received intragastric administration of amoxicillin (15 mg), metronidazole (15 mg), and clarithromycin (15 mg) mixture suspended in 1 mL of phosphate buffer once per day for three consecutive days); PRK1 (*n* = 20, rats were treated similarly to DCA group but were additionally daily dosed with a mixture of lyophilized *Lactobacillus acidophilus* (LA–5) and *Bifidobacterium animalis* subsp. *lactis (BB–12)* at a dose of 1.2 × 10^8^ colony-forming unit (CFU)/mL suspended in 1 mL of phosphate buffer for 15 days starting from the day 21); PRK2 (*n* = 20, rats were treated similarly to DCA group but were additionally daily dosed with 25 mg of *Saccharomyces boulardii* suspended in 1 mL of phosphate buffer for 15 days starting from the day 21); PRK3 (*n* = 20, rats were treated similarly to DCA group but were additionally daily dosed with *Enterococcus faecium L3* at a dose of 1.2 × 10^8^ CFU/mL suspended in 1 mL of phosphate buffer for 15 days starting from the day 21). After finalization of treatment with antimicrobial agents, the rats were recovered for 5 days before isolation of the heart. All the animals, except those in the CON group, were fed the HFCD throughout the period of recovery. The CON group received a standard diet (0 % sucrose, 12 % fat: Lab Diet, St. Louis, MO, USA) throughout the entire study. The following parameters were monitored: body weight, clinical status, as well as food and water intake at 9:00–10:00 a.m. each day during the 7 days prior to heart harvesting. After the autopsy, the cecum and the retroperitoneal, epididymal, and visceral fat pads were excised and weighed.

### 2.7. Assessment of Hematological, Biochemical, and Immunological Variables

The blood was taken from the saphenous vein for the analysis of hematological, immunological, and biochemical parameters using a standard blood collection technique five days after completion of treatment with antimicrobial agents in randomly selected animals (*n* = 10 in each group). A hematological automatic analyzer (ABX Micros 60, Horiba ABX, Montpellier, France) was used to assess hematological parameters such as red blood cells (RBC), white blood cells (WBC) and platelets (PLT). Centrifugation of the whole blood was repeated twice at 3000 rpm for 10 min to obtain serum.

Serum levels of total protein, triglycerides (TG), total cholesterol (CHOL), bile acids (BA), low-density lipoproteins (LDL), high-density lipoproteins (HDL), lactate, lactate dehydrogenase (LDH), alkaline phosphatase (ALP), urea, uric acid (URA), and creatinine (CRE) were determined using a biochemical analyzer (BioChem Analette; HTI, North Attleboro, MA, USA). The serum concentrations of interleukin (IL)–1β, tumor necrosis factor–α (TNF–α), IL–2, IL–8, IL–10, brain-derived neurotrophic factor (BDNF), transforming growth factor–β (TGF–β), and lipopolysaccharide (LPS) were measured using standard ELISA kits (MR–96A; Mindray, Shenzhen, China) according to the manuals of the manufacturer, and each ELISA test was repeated three times.

### 2.8. Analysis of Gut Microbiota

Determination of the gut microbiome components in fecal pellets was performed in two stages. At the beginning, DNA was extracted from the supernatants of fecal suspensions with the use of a DNA extraction kit (QIAamp DNA Stool Mini Kit; QIAGEN, Hilden, Germany) in a dry block heater (Termit; DNA–Technology LLC, Moscow, Russian), followed by incubation. Then, real-time polymerase chain reaction (PCR) was carried out using the reaction mixture Colonoflor–16 (Alpha–Lab, Saint-Petersburg, Russian) and the T100™ Thermal Cycler (Bio–Rad, Hercules, CA, USA). Melt curve analysis was carried out directly after amplification to identify the targeted PCR product, and then it was quantitatively assessed by spectrophotometry (NanoDrop ND–1000; Peqlab, Erlangen, Germany). The number of bacteria was presented as lgCFU/g.

### 2.9. Analysis of SCFAs

Evaluation of plasma SCFA levels was performed by gas chromatography with flame ionization detection (GC–FID). Measurements of the concentrations of the following SCFAs were performed: propionic, acetic, isovaleric, and isobutyric acids. The SCFAs were identified by characteristic mass fragment ions and their retention times using the regimen of selected ion monitoring on a GC–FID system (Agilent 7890A; Agilent Technologies, Wilmington, NC, USA). SCFA quantification was carried out by automatic integration of chromatograms using GC/MSD ChemStation software (Agilent Technologies, Wilmington, NC, USA).

### 2.10. Histopathological Studies

After autopsy, samples of the colon were pre-fixed in buffered 10 % Paraform, embedded in paraffin, cut into 5–µm sections, and stained with hematoxylin plus eosin (H&E) for histological evaluation using standard application methods. The slides were observed and photographed after H&E staining using a light microscope (DM750; Leica, Wetzlar, Germany) at 100× magnification. Analysis of the slides was performed by a pathologist who was blinded to the different treatment. The severity of CIC was quantified according to a scoring system proposed by Dieleman et al. [19].

### 2.11. Isolated Heart Perfusion and Infarct Size Measurement

The rats (*n* = 10 in each group) were anesthetized with isoflurane (5%). The hearts were removed via bilateral thoracotomy and placed in an ice-cold buffer. After that, the ascending aorta was cannulated, followed by initiation of perfusion with Krebs–Henseleit buffer composed of the following ingredients (in mmol/L): NaCl, 118.5; KCl, 4.7; NaHCO_3_, 25; KH_2_PO_4_, 1.2; MgSO_4_, 1.2; glucose, 11; and CaCl_2_, 1.5. Perfusion pressure was maintained at 85 mm Hg by gravity using a water-jacketed glass column coupled to the aortic cannula by a 3-way stopcock [20]. Buffer was continuously gassed with carbogen (95 % O_2_ plus 5 % CO_2_) in order to maintain pH at 7.4 ± 0.1.

Monitoring of the left ventricular systolic pressure (LVSP) and left ventricular end-diastolic pressure (LVEDP) was performed isovolumetrically using a thin-walled polyethylene balloon inserted into the left ventricle. The balloon was connected to an insulin syringe and inflated with 0.4–0.6 mL of water to obtain an LVEDP less than 10 mm Hg during stabilization. PhysExp Gold software (Cardioprotect Ltd., Saint Petersburg, Russian) was used to monitor the intraventricular pressure using a small transducer (Baxter International, Deerfield, IL, USA). Calculation of the left ventricular developed pressure (LVDP) was performed as the difference between LVSP and LVEDP. Heart rate (HR) was calculated from the pressure wave. Monitoring of the coronary flow rate (CFR) was performed by timed collection of perfusate in a graduated cylinder. After cannulation and initiation of perfusion, the hearts were stabilized for 15 min, and then subjected to 30-min global ischemia and 120-min reperfusion. Hemodynamic parameters were registered at 5 min prior to global ischemia and at the 15, 30, 45, 60, 75, 90, and 120th min of reperfusion. In addition, left ventricular pressure (LVP) was monitored at the 5, 10, 15, 20, 25, and 30th min of global ischemia. Next, exclusion criteria were applied: any heart with a HR less than 220 bpm and a CFR larger than 18 or less than 8 mL/min at the end of stabilization was excluded from the study. Moreover, hearts failing to generate an LVDP greater than 100 mm Hg at LVEDP of less than 10 mm Hg were also excluded.

After experiment, each heart was rapidly rinsed and cut into 5 equal transverse slices. The slices were incubated in a 1% solution of 2,3,5–triphenyltetrazolium chloride for 15 min at 37 °C. Stained slices were photographed by stereomicroscope (SMZ18; Nikon, Tokyo, Japan) connected to a digital camera (DS–Fi2, Nikon, Tokyo, Japan) for further analysis of the non-stained (infarcted) areas. Infarct size have been determined by computer-based planimetry using specific software (ImageJ 1.34s; National Institutes of Health (NIH) Bethesda, MD, USA). The algorithm included the application of cutoff value of color intensity derived from the mean intensities typical of non-ischemic and necrotic tissue. Infarct size was calculated as a percentage of surfaces of total ventricular area minus that of the cavities. The values for each slice were summarized and divided by the number of slices in order to obtain the value in a particular heart. 

### 2.12. Statistical Analysis

The data on body and organ weight, consumption of food and water, hematology, biochemistry and immunology, SCFA levels, hemodynamics and infarct size are presented as mean ± standard deviation (SD). The data on the number of gut microorganisms and histopathological scores are presented as box plots with whiskers (median and quartiles). Statistical analysis was carried out by SPSS 12.0 (IBM Corporation, Armonk, NY, USA). Analysis of the intra-group differences in hemodynamic values was performed using Friedman’s repeated-measures analysis of variance (ANOVA) on ranks test followed by Dunn’s multiple comparisons test. A post hoc test was performed only if the ANOVA analysis resulted in F less than 0.05 and there was no variance in homogeneity. Determination of the differences in infarct size, gut bacterial counts, blood counts, SCFA levels, biochemical and immunological parameters, and histological scores was performed by the Kruskal–Wallis test. Then the pairwise intergroup comparisons were performed with use of nonparametric Mann–Whitney U test. Statistical significance was defined as a *p* value of less than 0.05.

## 3. Results

### 3.1. Animal Body Weight, Consumption of Water and Food, and Organ Weight

**Figure 2:**
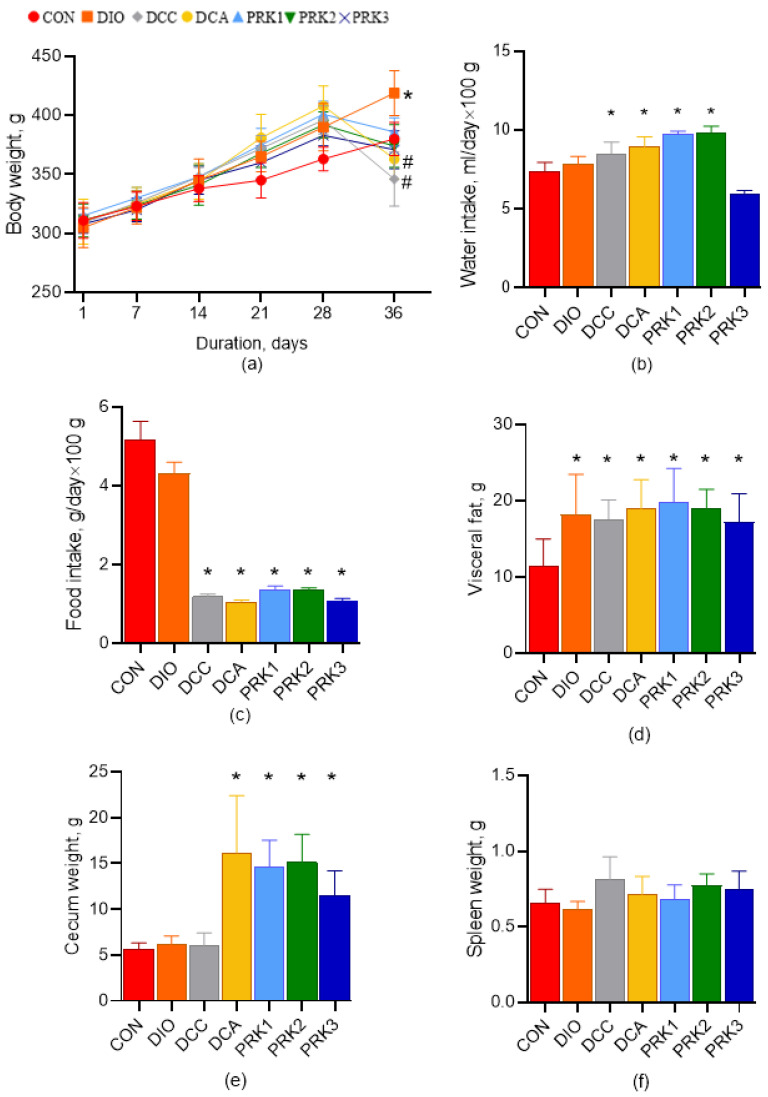
Effects of different treatments on animal body weight, food and water intake, and the weights of visceral fat, cecum and spleen. (**a**) Weekly changes in body weight; (**b**) Average water intake; (**c**) Average food intake; (**d**) Visceral fat weight; (**e**) Cecum weight; (**f**) Spleen weight. Data are expressed as mean ± SD. * indicates *p* < 0.05 when data is compared to that for the CON group; # indicates *p* < 0.05 when data is compared to that for the DIO group.

At the end of the study, animal body weight was significantly higher in the DIO group compared to the CON group (*p* < 0.05; Figure 2a). At the same time, body weight was significantly smaller at day 35 in DCC and DCA groups than in the DIO group (*p* < 0.05). There were no other intergroup differences in body weight. Water intake was higher in DCC, DCA, PRK1, and PRK2 groups compared to the CON group (*p* < 0.05; Figure 2b). Food intake has been found to be significantly lower in DCC, DCA, PRK1, PRK2, and PRK3 groups compared to the CON group (*p* < 0.05; Figure 2c). Visceral fat weight was higher in studies on all groups in comparison to the CON group (*p* < 0.05; Figure 2d). Caecum weight was significantly greater in DCA, PRK1, PRK2, and PRK3 groups versus the CON group (*p* < 0.05; Figure 2e). There were no differences in spleen weight between the groups (Figure 2f).

### 3.2. Hematological, Biochemical, and Immunological Parameters

White blood cell count was significantly higher in DCA and PRK3 groups in comparison to the CON group (*p* < 0.05; Table 1). In PKR1 group, leukocyte count was significantly smaller than in DCA group (*p* < 0.05). There were no differences in red blood cell counts between groups. Platelet count was higher in PRK2 and PRK3 groups compared to the CON group (*p* < 0.05; Table 1).

Among the biochemical parameters, we identified a significant decrease in BA levels in DCC, DCA, PRK2, and PRK3 groups versus the CON group (*p* < 0.05; Table 2). Interestingly, BA level in the PRK1 group was not different from the CON group but it was significantly higher than in DCA group (*p* < 0.05). Additionally, lactate levels were found to be higher in DCC, DCA, PRK1, PRK2, and PRK3 groups compared to the CON group (*p* < 0.05). LDH level was higher in DCA and PRK2 groups than in the CON group (*p* < 0.05). ALP activity was elevated in DIO, DCC, and DCA groups versus the CON group (*p* < 0.05). Administration of probiotics in PRK1, PRK2, and PRK3 groups resulted in normalization of ALP activity (*p* < 0.05 vs. DCA group). The level of urea was smaller in DCA and PRK3 groups compared to the CON group (*p* < 0.05). In PRK2 group, urea concentration was not different from that in the CON group, but it was significantly higher compared to DCA group (*p* < 0.05). URA was elevated in all studied groups than in the CON group (*p* < 0.05). CRE levels were significantly higher in DCA and PRK3 groups in comparison with the CON group (*p* < 0.05).

Systemic inflammation in DCC and DCA groups resulted in significantly elevated levels of IL–1β compared to the CON group (*p* < 0.05; Table 3). Probiotic treatment irrespective of the type of strain resulted in the normalization of IL–1β levels (*p* < 0.05 for PRK1, PRK2, and PRK3 groups vs. DCA group). TNF–α level was elevated in DCA group compared to the CON group (*p* < 0.05); administration of probiotics in PRK1, PRK2, and PRK3 groups caused normalization of TNF–α levels (*p* < 0.05 for PRK1, PRK2, and PRK3 groups vs. DCA group). Both IL–2 and IL–8 were significantly elevated in DIO, DCC, and DCA groups versus the CON group (*p* < 0.05). In PRK1 and PRK2 groups, IL–2 level was smaller compared to DCA group (*p* < 0.05) and was not different from that in the CON group. However, IL–2 concentration in PRK3 group was significantly different from both CON and DCA groups (*p* < 0.05) meaning that treatment with *Enterococcus faecium L3* resulted in incomplete normalization of IL–2 level. Normalization of IL–8 levels was registered in PRK1 and PRK3 groups only (*p* < 0.05 vs. DCA group). BDNF level was significantly elevated in DCC, DCA, and PRK3 groups in comparison to the CON group (*p* < 0.05). In the PRK1 group, BDNF level was smaller than in DCA group (*p* < 0.05). Among anti-inflammatory cytokines, we found elevation of TGF–β in DCC, DCA, and PRK2 groups compared to the CON group (*p* < 0.05). In PRK3 group, TGF–β level was significantly lower than in DCA group (*p* < 0.05). The levels of IL–10 were not different among groups. Finally, LPS level was dramatically higher in DIO, DCC, DCA, PRK2, and PRK3 groups in comparison to the CON group (*p* < 0.05). Normalization of LPS level was observed in the PRK1 group only (*p* < 0.05 vs. DCA group).

### 3.3. Alterations in Gut Microbiota

**Figure 3:**
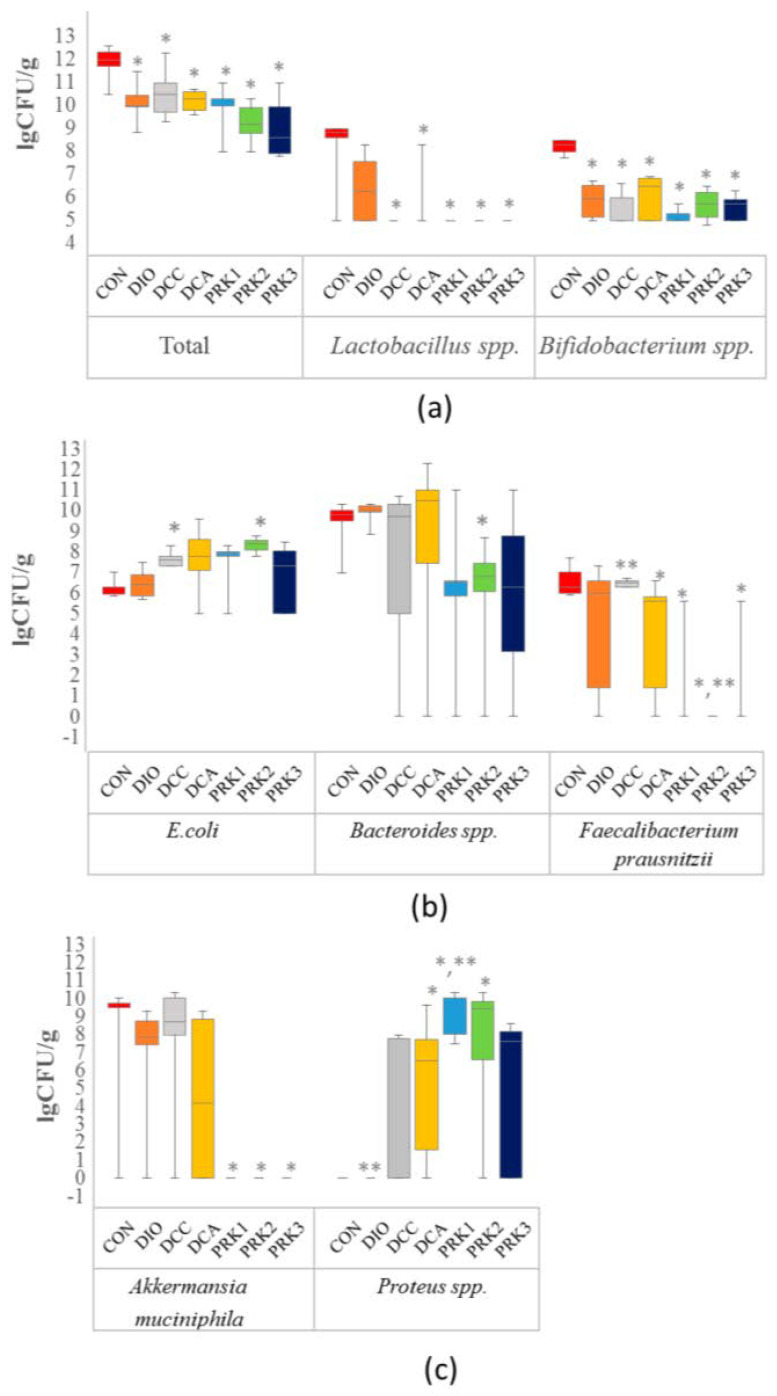
Effects of different treatments on the composition of intestinal microbiome. Bacterial counts were analyzed in fecal samples obtained at the end of the experiments using RT–PCR. (**a**) Total bacterial count, *Lactobacillus* spp., *Bifidobacterium* spp.; (**b**) *E. coli, Bacteroides* spp., *Faecalibacterium prausnitzii*; (**c**) *Akkermansia muciniphila, Proteius* spp. Data are expressed presented as box plots with median and quartiles as well as whiskers to express minimum and maximum values. * indicates *p* < 0.05 when data is compared to that for the CON group; ** indicates *p* < 0.05 when data is compared to that for the DCA group.

Total bacterial count was significantly lower in all studied groups than in the CON group (*p* < 0.05, Figure 3a). Additionally, *Lactobacillus* spp. and *Bifidobacterium* spp. counts were found to be reduced in all groups relative to the CON group (*p* < 0.05, Figure 3a). The animals in DCC and PRK2 groups had a higher count of E. coli than those in the CON group did (*p* < 0.05, Figure 3b). *Bacteroides* spp. count was significantly reduced in PRK2 group in comparison to the CON group (*p* < 0.05, Figure 3b). The count of *Faecalibacterium prausnitzii* was significantly reduced in DCA, PRK1, PRK2, and PRK3 groups versus the CON group (*p* < 0.05, Figure 3b). Furthermore, *Faecalibacterium prausnitzii* count was significantly lower in PRK2 group compared to DCA group. *Akkermansia muciniphila* count was reduced in all groups treated with different probiotics as compared to the CON group (*p* < 0.05, Figure 3c). *Proteus* spp. population was elevated in DCA, PRK1, and PRK2 groups compared to the CON group (*p* < 0.05, Figure 3c). In addition, *Proteus* spp. count was significantly greater in the PRK1 group than in the DCA group (*p* < 0.05).

### 3.4. Short-Chain Fatty Acids

**Figure 4:**
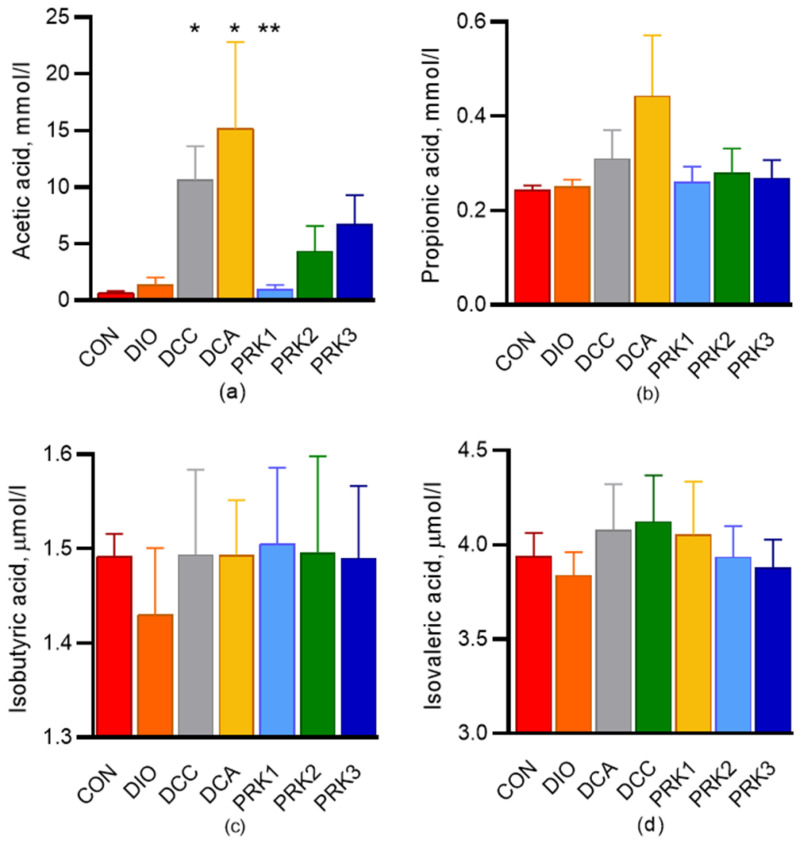
Effects of different treatments on plasma concentration of short-chain fatty acids. Plasma concentrations of (**a**) acetic, (**b**) propionic, (**c**) isobutyric, and (**d**) isovaleric acid were determined by GC–FID. Data are presented as mean ± standard deviation. * indicates *p* < 0.05 when data is compared to that for the CON group; ** indicates *p* < 0.05 when data is compared to that for the DCA group.

Serum levels of acetic, propionic, isobutyric, and isovaleric SCFAs at the end of the experiment are shown in Figure 4. Serum acetate level was significantly higher in the DCC and DCA groups in comparison with the CON group (*p* < 0.05, Figure 4a). In the PRK1 group, acetate concentration was not different from the controls; furthermore, it was significantly lower than in DCA group (*p* < 0.05, Figure 4a). Propionate level tended to be higher in the DCA group compared to the CON group, but the difference was not statistically significant. The levels of isobutiric and isovaleric acids were not different among groups (Figure 4c,d). 

### 3.5. Hemodynamic Parameters of the Isolated Heart and Myocardial Infarct Size

**Figure 5:**
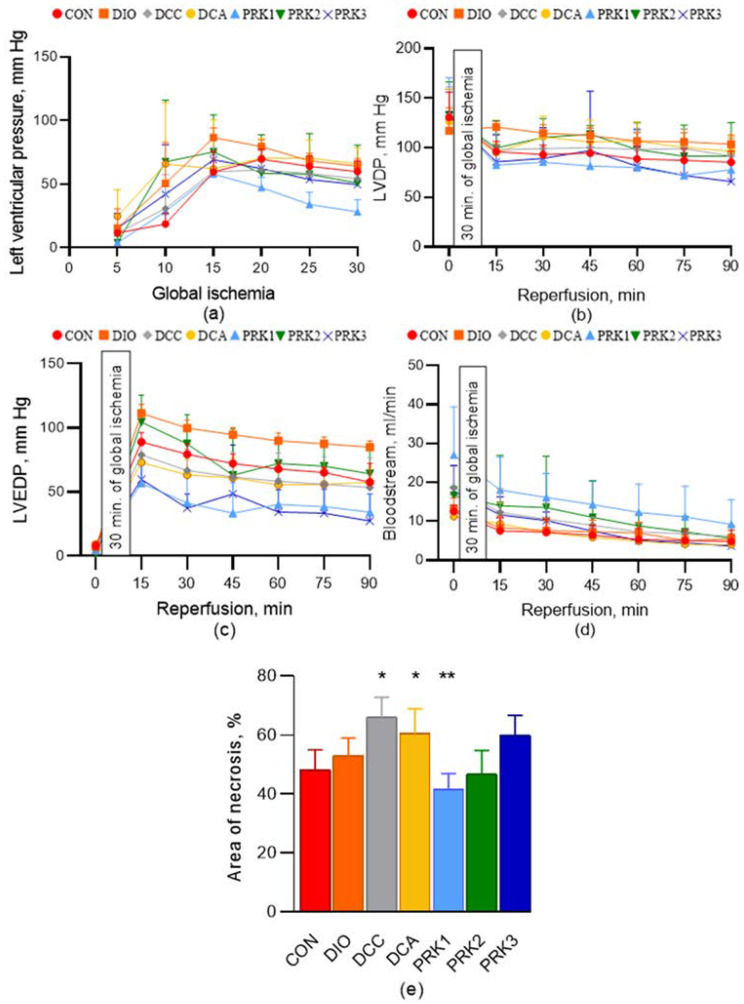
Hemodynamic parameters and necrosis (infarct) size in isolated Langendorff-perfused rat hearts subjected to 30 min of global ischemia and 120 min of reperfusion. (**a**) Ischemic contracture, (**b**) LVDP, (**c**) LVEDP, (**d**) coronary flow rate, and (**e**) area of necrosis. Data are presented as mean ± standard deviation. * indicates *p* < 0.05 when data is compared to that for the CON group; ** indicates *p* < 0.05 when data is compared to that for the DCA group.

The values of LVP during the period of 30-min global ischemia are shown in Figure 5a. The initial LVDP, LVEDP, and coronary flow rate values were not different among groups (Figure 5b–d). No intergroup differences in hemodynamic parameters over the 90–min reperfusion period were observed. The heart rate values (also not different among groups) are provided in Table S1.

Myocardial infarct size was 49 ± 8% in the CON group (Figure 5e). Infarct size was not different from the controls in the DIO group (53 ± 7%, *p* > 0.05). In DCC and DCA groups, infarct size was significantly larger than in the CON group (66 ± 7 and 60 ± 8 %, *p* < 0.05). Importantly, infarct size was significantly smaller in the PRK1 group compared to that in the DCA group (41 ± 5%, *p* < 0.05). It also tended to be lower than in the CON group, but this difference has not been proved statistically. Infarct size in PRK2 and PRK3 groups was not different from either the CON group or the DCA group.

### 3.6. Histopathological Data 

**Figure 6:**
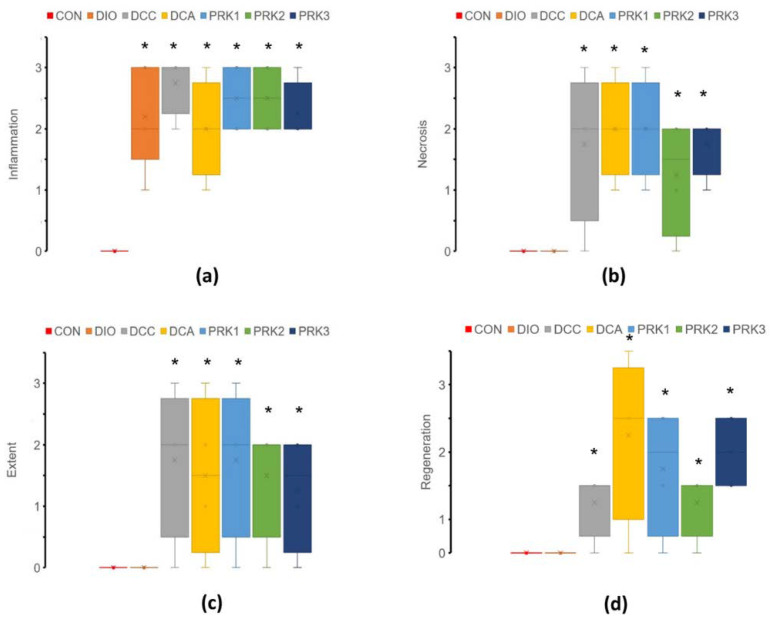
Histopathological colitis scores. (**a**) Inflammation; (**b**) necrosis; (**c**) extent; (**d**) regeneration. Data are expressed presented as box plots with median and quartiles as well as whiskers to express minimum and maximum values. * indicates *p* < 0.05 when data is compared to that for the CON group.

Colon histology was normal in the CON group (Figure 6). DIO group was characterized by significantly elevated inflammation score compared to the CON group, while other scores were not changed. Such scores as inflammation, necrosis, extent, and regeneration were all significantly higher in DCC, DCA, PRK1, PRK2, and PRK3 groups versus the CON group (*p* < 0.05).

## 4. Discussion

One of the main findings of the present study was that the intragastric administration of lyophilized *Lactobacillus acidophilus* and *Bifidobacterium animalis* subsp. *lactis* at a dose of 1.2 × 10^8^ CFU/mL for 15 days resulted in myocardial infarct size reduction in rats with DIO, CIC, and antibiotic-induced dysbiosis.

Accumulating evidence suggests that the infarct-limiting effect of certain probiotics and antibiotics might occur due to altered composition of intestinal microbiome, although the direct effects of the absorbed compounds on cardiomyocytes are not excluded. In the first study demonstrating the effect of changed intestinal microbiome on myocardial IRI, it has been shown that either *L. plantarum 299V* or vancomycin administration was associated with plasma leptin level reduction and infarct size limitation [4]. Notwithstanding, our recent study showed that 8–day hyperleptinemia in rats is associated with exaggerated myocardial IRI, elevated proinflammatory cytokines, and adverse myocardial remodeling after infarction [21]. Initial observations with vancomycin-induced cardioprotection have been subsequently confirmed in a study showing infarct size limitation in rats after administration of a mixture of streptomycin, bacitracin, polymixin B, and neomycin [5]. Of note, antibiotic-induced cardioprotection has been associated with specific changes in gut microbiome, which was confirmed by metabolomics analysis. In this study, we intended to investigate the relationship between probiotic-induced cardioprotection, gut microbiota patterns, plasma SCFA levels, and pro-/anti-inflammatory cytokines. The effects of three different probiotics on myocardial infarct size were analyzed in the animals with comorbidity, which is critical for improved translatability of the results to the clinical practice [22]. In order to overcome translational barriers in cardioprotection, current guidelines strongly recommend testing new cardioprotective interventions in aged animals with comorbidities [23]. In the present study, we used a previously validated rat model of DIO aggravated by systemic inflammatory response syndrome (SIRS) and antibiotic-induced dysbiosis. DIO was elicited by HFCD and confirmed using such criteria as body weight and visceral fat gain. Literature data on the effects of DIO on myocardial IRI are contradictory. Some studies demonstrated that high-fat diet-induced obesity in animals is associated with greater infarct size, which is in a good agreement with clinical data [24]. Contrary to that, other authors have reported attenuated myocardial IRI in animals with HFCD-induced obesity, an effect which has been attributed to more gradual restoration of pH within cardiac myocytes in the reperfusion period [25]. In our experiments, DIO was not associated with changes in infarct size, although some deviations in the microbiome composition and cytokine levels have been detected in this group.

The presence of CIC was verified in this study with the use of histological scores. In addition, CIC development was confirmed by significant elevation of proinflammatory cytokines, weight loss, decreased food consumption and increased water intake. Importantly, CIC and associated SIRS resulted in significantly greater infarct size compared to controls and DIO group. This might be accounted for by elevated proinflammatory cytokines, such as IL–1β, IL–2, and IL–8, which are known mediators of myocardial dysfunction and cardiomyocyte apoptosis [26]. This hypothesis is further strengthened by recent evidence on cardioprotective effects of IL–1 receptor type 2, playing a role of soluble decoy receptor of IL–1β [27]. A similar effect has been demonstrated using monoclonal anti-TNF–α antibodies [28].

The main hypothesis of the present study was that supplementation of intestinal microbiome with exogenous probiotics could “optimize” the composition of microflora, thereby resulting in systemic neurohumoral changes responsible for infarct size limitation. Although all three different probiotics normalized to a variable extent the alterations in biochemical and immunological parameters elicited by DIO, SIRS, and antibiotic-induced dysbiosis, a significant infarct-limiting effect was found only in the PRK1 group. Of note, there were no specific changes in microbiome unique to the PRK1 group, at least in the bacterial genera analyzed. One might suggest that the putative cardioprotective effect of PRK1 is attributed to better healing of the mucosal injury in the colon. Again, this is not the case, because neither probiotic resulted in significant improvement of histopathological scores characterizing colitis severity. Among putative differentially expressed in the PRK1 group cardioprotective metabolites, we focused on the concentrations of SCFAs. The only significant change observed was the reduction in acetate level in the PRK1 group compared to the DCA group. However, it is unlikely that this change plays a causative role in PRK1-induced cardioprotection. It is known that propionate is the major ligand for free fatty acid receptor (FFAR)–3 (FFAR3). In fact, elevated propionate signaling through FFAR3 both in cardiac myocytes and in neuronal endings has been recently suggested as a mechanism of myocardial injury and adverse remodeling [29]. Therefore, it might be anticipated that the blockers of FFAR3 would demonstrate cardioprotective properties, a hypothesis that requires rigorous testing. Obviously, probiotic treatment might be associated with changes in a number of other metabolites. One promising target is TGR5 receptor, which is activated by bile acids. Recent evidence suggests that the activation of TGR5 receptor by deoxycholic acid results in reduced inflammation and improved cardiac function in myocardial infarction [30].

## 5. Conclusions

In conclusion, the intragastric administration of lyophilized *Lactobacillus acidophilus* and *Bifidobacterium animalis* subsp. *lactis* at a dose of 1.2 × 10^8^ CFU/mL for 15 days resulted in myocardial infarct size reduction in rats with DIO, CIC, and antibiotic-induced dysbiosis. This cardioprotective effect was associated with specific changes in cytokine concentrations, namely, reduced levels of IL–1β, TNF–α, IL–2, and IL–8. At the same time, the use of *Lactobacillus acidophilus* and *Bifidobacterium animalis* subsp. *lactis* was accompanied by a significant reduction in LPS level, suggesting normalization of intestinal epithelial barrier permeability. However, the cardioprotective effect of *Lactobacillus acidophilus* and *Bifidobacterium animalis* subsp. *lactis* is not secondary to improved healing of the intestinal mucosa in CIC, as evidenced by the lack of difference in histopathological scores.

## Figures and Tables

**Table 1:** Peripheral blood cell counts in Wistar rats at the end of the experiments (*n* = 10 in each group). *—*p* < 0.05 vs. CON group; **—*p* < 0.05 vs. DCA group. WBC—white blood cells, RBC—red blood cells, PLT—platelets. Group names are specified in the text.

	CON	DIO	DCC	DCA	PRK1	PRK2	PRK3
WBC (×10^9^/L)	5.1 ± 0.7	5.5 ± 0.4	6.8 ± 1.3	7.1 ± 2.2 *	5.2 ± 0.9 **	5.9 ± 1.9	8.8 ± 1.6 *
RBC (×10^12^/L)	7.4 ± 0.5	7.1 ± 0.4	7.3 ± 0.6	7.5 ± 0.3	7.3 ± 0.3	7.1 ± 1.2	7.2 ± 1.1
PLT (×10^9^/L)	475 ± 52	505 ± 66	598 ± 130	548 ± 212	557 ± 99	651 ± 95 *	618 ± 108 *

**Table 2:** Biochemical serum markers in Wistar rats at the end of the experiment (*n* = 10 in each group). The results show mean values ± SD. *—*p* < 0.05 vs. CON group; **—*p* < 0.05 vs. DCA group. TG—triglycerides; CHOL—total cholesterol; BA—bile acids; LDL—low-density lipoproteins; HDL—high-density lipoproteins; LDH—lactate dehydrogenase; ALP—alkaline phosphatase; URA—uric acid; CRE—creatinine.

	CON	DIO	DCC	DCA	PRK1	PRK2	PRK3
Protein (g/L)	64 ± 3	62 ± 6	61 ± 4	63 ± 2	64 ± 3	65 ± 3	63 ± 3
TG (mg/dL)	74 ± 31	79 ± 38	72 ± 35	76 ± 41	91 ± 37	84 ± 35	97 ± 45
CHOL (mg/dL)	71 ± 36	76 ± 49	71 ± 35	74 ± 38	76 ± 41	69 ± 39	72 ± 37
BA (μM/L)	12.1 ± 4.4	10.8 ± 3.9	1.6 ± 1.5 *	1.8 ± 1.2 *	10.9 ± 3.2 **	4.3 ± 3.5 *	0.7 ± 0.3 *
LDL (mg/dL)	26 ± 4	23 ± 6	24 ± 4	20 ± 4	26 ± 7	26 ± 8	23 ± 7
HDL (mg/dL)	49 ± 5	46 ± 7	39 ± 6	49 ± 2	46 ± 10	43 ± 5	41 ± 6
Lactate (mg/dL)	42 ± 7	46 ± 14	65 ± 10 *	68 ± 14 *	67 ± 10 *	69 ± 9 *	64 ± 5 *
LDH (U/L)	623 ± 130	1027 ± 314	979 ± 387	1183 ± 223 *	929 ± 395	1250 ± 264 *	1022 ± 334
ALP (U/L)	21 ± 4	41 ± 10 *	54 ± 13 *	57 ± 14 *	31 ± 9 **	27 ± 12 **	25 ± 6 **
Urea (mg/dL)	15 ± 1	16 ± 3	13 ± 2	11 ± 1 *	12 ± 3	15 ± 2 **	11 ± 2 *
URA (μM/L)	44 ± 10	88 ± 32 *	84 ± 41 *	76 ± 15 *	81 ± 19 *	78 ± 13 *	85 ± 16 *
CRE (μM/L)	8 ± 2	13 ± 3	11 ± 3	15 ± 4 *	12 ± 2	11 ± 2	17 ± 3 *

**Table 3:** Serum concentrations of cytokines, brain-derived neurotrophic factor, and lipopolysaccharide in Wistar rats at the end of the experiment (*n* = 10 in each group). The results show mean values ± SD. *—*p* < 0.05 vs. CON group; **—*p* < 0.05 vs. DCA group. IL–1β—interleukin–1β; TNF–α—tumor necrosis factor–α; IL–2—interleukin–2; IL–8—interleukin–8; BDNF—brain-derived neurotrophic factor; TGF–β—transforming growth factor–β; IL–10—interleukin–10; LPS—lipopolysaccharide.

	CON	DIO	DCC	DCA	PRK1	PRK2	PRK3
IL–1β (pg/mL)	51 ± 16	78 ± 29	99 ± 13 *	76 ± 12 *	45 ± 7 **	57 ± 10 **	56 ± 6 **
TNF–α (pg/mL)	19 ± 2	21 ± 5	21 ± 2	28 ± 3 *	20 ± 2 **	18 ± 2 **	17 ± 2 **
IL–2 (pg/mL)	2.4 ± 0.2	4.3 ± 0.6 *	6.7 ± 1.9 *	6.1 ± 1.4 *	2.9 ± 0.5 **	3.0 ± 0.7 **	3.9 ± 0.9 *^,^**
IL–8 (pg/mL)	28 ± 6	48 ± 9 *	72 ± 24 *	62 ± 12 *	32 ± 14 **	61 ± 28	25 ± 4 **
BDNF (ng/mL)	6 ± 2	7 ± 2	12 ± 4 *	9 ± 2 *	6 ± 2 **	7 ± 3	9 ± 3 *
TGF–β (ng/mL)	5 ± 2	7 ± 4	9 ± 3 *	12 ± 3 *	8 ± 4	16 ± 7 *	4 ± 2 **
IL–10 (pg/mL)	19 ± 2	23 ± 3	22 ± 3	18 ± 2	23 ± 2	19 ± 3	19 ± 3
LPS (ng/mL)	6 ± 2	11 ± 2 *	78 ± 10 *	32 ± 8 *	11 ± 5 **	36 ± 7 *	29 ± 2 *

## Data Availability

The data presented in this study are available on request from the corresponding author.

## Supplementary Materials

The following supporting information can be downloaded at: https://www.mdpi.com/article/10.3390/microorganisms10112293/s1, Table S1: The values of heart rate (beats/min) in the experimental groups. Data are mean ± SD. Group legends: controls (CON), diet-induced obesity (DIO), diet-induced obesity + chemically induced colitis (DCC), diet-induced obesity + chemically induced colitis + antibiotic-induced dysbiosis (DCA), diet-induced obesity + chemically induced colitis + antibiotic-induced dysbiosis + treatment with *Lactobacillus acidophilus (LA–5)* and *Bifidobacterium animalis* subsp. *lactis (BB–12)* (PRK1), diet-induced obesity + chemically induced colitis + antibiotic-induced dysbiosis + treatment with *Saccharomyces boulardii* (PRK2), diet-induced obesity + chemically induced colitis + antibiotic-induced dysbiosis + treatment with *Enterococcus faecium L3* (PRK3).
