# Supplementary material for: Probiotic Therapy with Lactobacillus acidophilus and Bifidobacterium animalis subsp. lactis Results in Infarct Size Limitation in Rats with Obesity and Chemically Induced Colitis
Source: Microorganisms. 2022 Nov 18;10(11):2293. doi: 10.3390/microorganisms10112293 (PMC9698902; doi:10.3390/microorganisms10112293)
Supplement: Supplementary file 1 [file microorganisms-10-02293-s001.zip › Table S1.pdf]

Table S1. The values of heart rate (beats/min) in the experimental groups. Data are mean ± SD. Group legends: Group legends: controls (CON), diet-induced obesity (DIO), diet-induced obesity + chemically induced colitis (DCC), diet-induced obesity + chemically induced colitis + antibiotic-induced dysbiosis (DCA), diet-induced obesity + chemically induced colitis + antibiotic-induced dysbiosis + treatment with *Lactobacillus acidophilus (LA–5)* and *Bifidobacterium animalis subsp. lactis (BB–12)* (PRK1), diet-induced obesity + chemically induced colitis + antibiotic-induced dysbiosis + treatment with *Saccharomyces boulardii* (PRK2), diet-induced obesity + chemically induced colitis + antibiotic-induced dysbiosis + treatment with *Enterococcus faecium L3* (PRK3).

Groups	Baseline	Reperfusion						
		15 min	30 min	45 min	60 min	75 min	90 min	120 min
CON	366± 85	414 ± 38	369 ± 83	338 ± 72	298 ± 19	297 ± 22	307 ± 26	295 ± 25
DIO	444 ± 72	430 ± 57	364 ± 68	367 ± 74	357 ± 39	351 ± 56	403 ± 111	387 ± 84
DCC	401 ± 92	398 ± 53	384 ± 135	358 ± 79	359 ± 87	373 ± 86	364 ± 74	352 ± 53
DCA	370 ± 124	385 ± 59	352 ± 85	363 ± 47	401 ± 76	391 ± 79	411 ± 95	385 ± 76
PRK1	391 ± 109	401 ± 64	353 ± 83	366 ± 76	351 ± 82	384 ± 62	398 ± 81	376 ± 65
PRK2	461 ± 105	435 ± 99	364 ± 104	411 ± 91	410 ± 99	413± 96	402 ± 97	395 ± 71
PRK3	416 ± 126	414 ± 109	421 ± 141	392 ± 97	397 ± 105	399 ± 59	406 ± 102	389 ± 75
